# A trypsin-like serine protease is involved in pseudorabies virus invasion through the basement membrane barrier of porcine nasal respiratory mucosa

**DOI:** 10.1186/1297-9716-42-58

**Published:** 2011-04-14

**Authors:** Sarah Glorieux, Herman W Favoreel, Lennert Steukers, Annelies P Vandekerckhove, Hans J Nauwynck

**Affiliations:** 1 Laboratory of Virology, Faculty of Veterinary Medicine, Ghent University, Salisburylaan 133, B-9820 Merelbeke, Belgium; 2 Laboratory of Immunology, Faculty of Veterinary Medicine, Ghent University, Salisburylaan 133, B-9820 Merelbeke, Belgium

## Abstract

Several alphaherpesviruses breach the basement membrane during mucosal invasion. In the present study, the role of proteases in this process was examined. The serine protease-specific inhibitor AEBSF inhibited penetration of the basement membrane by the porcine alphaherpesvirus pseudorabies virus (PRV) by 88.1% without affecting lateral spread. Inhibitors of aspartic-, cysteine-, and metalloproteases did not inhibit viral penetration of the basement membrane. Further analysis using the Soybean Type I-S trypsin inhibitor for the serine protease subcategory of trypsin-like serine proteases resulted in a 96.9% reduction in plaque depth underneath the basement membrane. These data reveal a role of a trypsin-like serine protease in PRV penetration of the basement membrane.

## Introduction, Methods and Results

The basement membrane (BM) constitutes part of the extracellular matrix and creates a formidable barrier to invading pathogens [1]. Crossing the BM may facilitate viral access to blood vessels and nerves in the lamina propria whereafter it may spread to internal organs. Following infection of respiratory epithelial cells, different alphaherpesviruses are able to cross the BM facilitating viral invasion in the body and leading to viremia, virus dissemination and aggravated general clinical signs [2-12]. Using porcine nasal respiratory mucosal explants, we have previously confirmed that the porcine alphaherpesvirus pseudorabies virus (PRV) efficiently breaches the BM [3]. The underlying mechanism of herpesvirus passage across the BM is unknown.

Breach of the BM in disease states is best characterized in metastatic progression in oncology following neoplastic establishment and often involves disruption of the BM by proteolytic enzymes [13,14]. Therefore, in the current study, we investigated whether proteases are involved in alphaherpesvirus invasion through the BM. We reported previously an in vitro model that enables study and quantitative analysis of PRV invasion through the BM in nasal respiratory mucosa [3,15]. Porcine nasal respiratory explants were obtained as described previously [15]. Briefly, explants were stripped from the surfaces of ventral turbinates and septum, and incubated with the epithelial surface upwards on fine-meshed gauze and cultured at the air-liquid interface with serum-free medium (50% RPMI (Invitrogen, Paisley, UK)/50% DMEM (Invitrogen) supplemented with 1 μg/mL gentamycin (Invitrogen), 0.3 mg/mL glutamin (VWR, West Chester, PA, USA), 0.1 mg/mL streptomycin (Certa, Braine l'Alleud, Belgium) and 100 U/mL penicillin (Continental Pharma, Puurs, Belgium)). Explants were cultivated for 10 h before inoculation with 600 μL medium containing 10^7 ^TCID_50 _of PRV field strain 89V87 [16]. After incubation for 1 h, explants were washed three times with serum-free medium.

Proteases are classified according to their catalytic activity: serine-, cysteine-, metallo- and aspartic peptidases [17,18]. The effect of inhibition of these protease types on PRV penetration through the BM was investigated using Complete Mini Protease Inhibitor Cocktail Tablets containing a proprietary mixture of several protease inhibitors with broad inhibitory specificity for serine, cysteine, and metalloproteases (Roche Diagnostics Corporation, Basel, Switzerland). To investigate the involvement of aspartyl proteases, pepstatin A (Sigma, St. Louis, MO, USA), was used. Inhibitor concentrations were used as recommended by the manufacturer's instruction, one tablet complete Mini protease inhibitor cocktail per 10 mL and 1 μg/mL pepstatin A. At 1 h post inoculation (pi), medium was replaced by medium with and without inhibitor for inhibitor-treated and mock-treated explants respectively. Explants were immersed for 1 h and then transferred again to the gauze and cultivated with medium in the presence or absence of inhibitor for inhibitor-treated and mock-treated explants respectively until sampling. Samples were collected at 20 h pi, embedded in methocel^® ^(Sigma) and frozen at -70°C. The time point of sampling was specified at 20 h pi because PRV was found to cross the BM between 12 and 16 h pi (data not shown). Cryosections were made, fixed in methanol, stained for collagen IV (BM component) and PRV and analyzed by confocal microscopy. Viral invasion across and lateral spread above the BM were analyzed using ImageJ, as reported previously [3]. For each condition, maximal plaque latitude and depth underneath the BM were measured for 10 plaques; triplicate independent experiments were performed.

**Figure 1:**
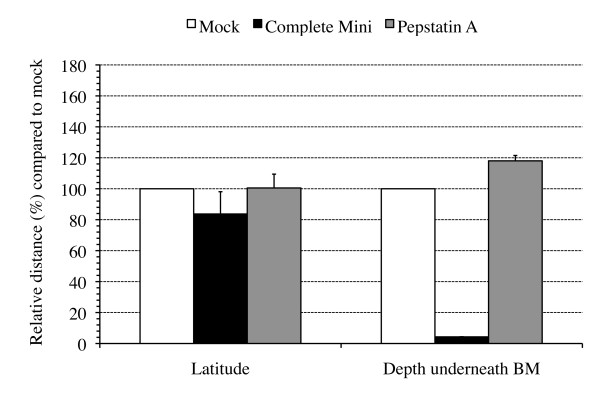
**Plaque latitude and penetration depth underneath the basement membrane (BM) of PRV(89V87) plaques at 20 h pi in mock-treated (white bars) and protease inhibitor-treated (marked bars) porcine nasal respiratory mucosa explants**. Explants were treated with a broad-spectrum protease inhibitor cocktail (complete Mini), inhibiting serine, cysteine and metalloproteases, or with an aspartyl protease inhibitor, pepstatin A, at 1 h pi until sampling. Data are represented as means of 10 plaques of duplicate independent experiments + SD (error bars).

Figure 1 shows mean values + SD of duplicate independent experiments for PRV invasion across and lateral spread above the BM. Incubation of PRV-inoculated explants with the serine-, cysteine- and metalloprotease inhibitor cocktail resulted in a 94.9% reduction in distance covered underneath the BM. The plaque latitude remained similar, indicating that the inhibitor did not affect viral replication in general. Pepstatin A did not reduce plaque depth underneath the BM. These results suggest the involvement of a serine-, cysteine- and/or metalloprotease in PRV invasion through the BM.

**Figure 2:**
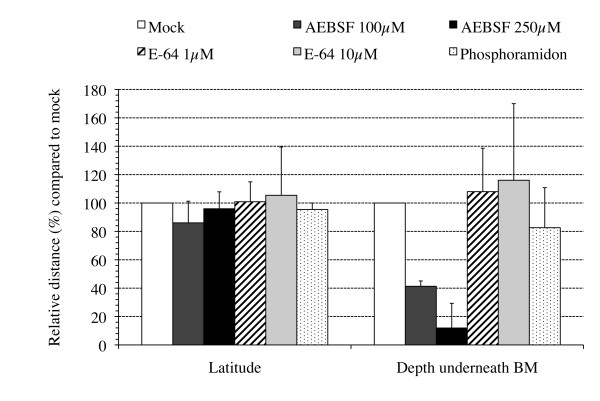
**Plaque latitude and penetration depth underneath the basement membrane (BM) of PRV(89V87) plaques at 20 h pi in mock-treated (white bars) and protease inhibitor-treated (marked bars) porcine nasal respiratory mucosa explants**. Explants were treated with 100 or 250 μM AEBSF, 1 or 10 μM E-64 or 10 μM phosphoramidon at 1 h pi until sampling. Data are represented as means of 10 plaques of triplicate independent experiments + SD (error bars).

To further delineate which of the serine-, cysteine- and/or metalloproteases are involved in PRV invasion through the BM, type-specific inhibitors were used: AEBSF (4-(2-Aminoethyl) benzenesulfonyl fluoride hydrochloride) inhibits serine proteases, E-64 (trans-Epoxysuccinyl-l-leucylamido-(4-guanidino)butane) inhibits cysteine proteases and phosphoramidon inhibits metalloproteases (Sigma). PRV-inoculated explants were treated with 100 or 250 μM AEBSF, 1 or 10 μM E-64 or 10 μM phosphoramidon and the effect on the plaque depth underneath the BM and the plaque latitude above the BM was evaluated. Figure 2 shows a dose-dependent decrease in the plaque depth underneath the BM when using AEBSF at 100 and 250 μM where the inhibitor reduced plaque depth underneath the BM by 58.7 and 88.1% respectively, compared with plaques from mock-treated explants. E-64 and phosphoramidon did not reduce plaque depth or latitude. These results suggest a role for serine proteases in PRV invasion across the BM.

**Figure 3:**
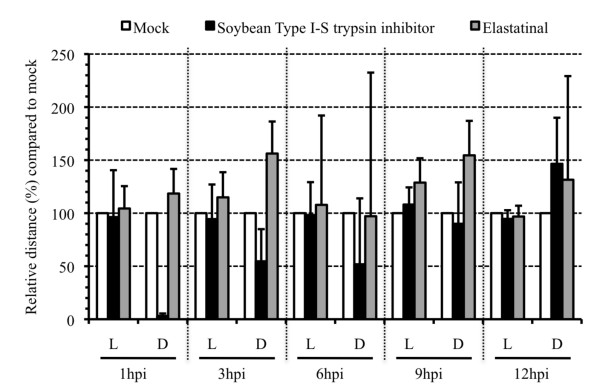
**Plaque latitude (L) and penetration depth underneath the basement membrane (D) of PRV(89V87) plaques at 20 h pi in mock-treated (white bars) and protease inhibitor-treated (marked bars) porcine nasal respiratory mucosa explants**. Explants were mock-treated or treated with a trypsin-like serine protease inhibitor, Soybean Type I-S trypsin inhibitor, or with an elastase-like serine protease inhibitor, elastatinal, at 1 h pi, 3 h pi, 6 h pi, 9 h pi or 12 h pi until sampling. Data are represented as means of plaques observed in 120 sections (20 μm) of triplicate independent experiments + SD (error bars).

**Figure 4:**
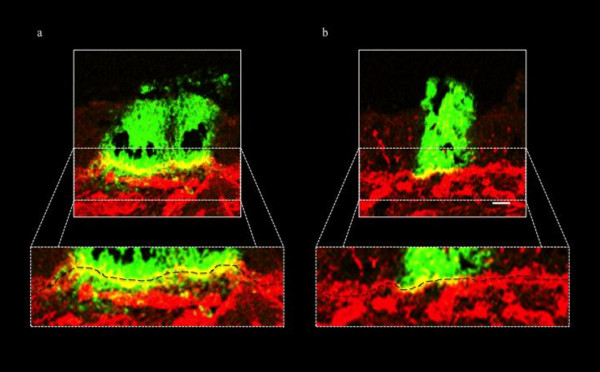
**Confocal photomicrographs illustrating PRV(89V87) plaques at 20 h pi in (a) mock-treated and (b) Soybean Type I-S trypsin inhibitor-treated porcine nasal respiratory mucosa explants**. Green fluorescence visualizes PRV antigens in virus plaques using FITC-conjugated polyclonal PRV-specific antibodies. Collagen IV (component of basement membrane (BM), black dashed line) is visualized using goat anti-collagen IV antibodies, stained afterwards with TexasRed. Figure (a) shows a representative image of two closely apposed viral plaques both invading through the BM. Figure (b) illustrates the blocked viral invasion underneath the BM in the presence of a trypsin-like protease inhibitor. Bar, 20 μm.

Based on their substrate specificity, serine proteases are generally classified into three main categories: trypsin-like, chymotrypsin-like and elastase-like serine proteases [19]. The trypsin inhibitor from *Glycine max*, Soybean Type I-S, was first isolated by Kunitz [20] and inhibits trypsin-like serine proteases (Sigma). TPCK (N-*p*-Tosyl-L-phenylalanine chloromethyl keton) inhibits chymotrypsin-like serine proteases and elastatinal is an inhibitor of elastase-like serine proteases (Sigma) [21]. A time course of protease inhibitor treatment was performed for these three inhibitors. At different time points pi, PRV-inoculated explants were either mock-treated or treated with 5 mg/mL Soybean Type I-S, 100 μM TPCK or 100 μM elastatinal [21]. Treatment started at 1 h pi, 3 h pi, 6 h pi, 9 h pi or 12 h pi until sampling at 20 h pi. To this end, explants were immersed for 1 h in medium with and without inhibitor at 1 h pi, 3 h pi, 6 h pi, 9 h pi or 12 h pi for inhibitor- and mock-treated explants respectively. After 1 h immersion, explants were transferred to the gauze and cultivated with medium with and without inhibitor for inhibitor- and mock-treated explants respectively until sampling. A wash-out experiment with each of the inhibitors was also performed. Therefore, inhibitor was added at 1 h pi, explants were immersed for 1 h, washed three times, transferred again to the gauze and cultivated with medium without inhibitor. For each protease inhibitor/time point combination, 120 cryosections (20 μm) were made. Figure 3 gives the mean values of the plaque latitude and depth underneath the BM of triplicate independent time course experiments. Inhibition of trypsin-like serine protease activity strongly reduced the plaque depth underneath the BM with 96.9% (illustrated in Figure 4) when added at 1 h pi until sampling and with 45.4, 48.2, 10.0% when added starting from 3, 6 and 9 h pi respectively until sampling. No reduction was observed when the inhibitor was added starting from 12 h pi. Treating the inoculated explants with inhibitor at 1 h pi and washing out the inhibitor at 2 h pi resulted in a 23.4% reduction in plaque depth underneath the BM (data not shown). Plaque latitudes always remained similar. No plaques were observed after treatment of PRV-inoculated explants with TPCK, except for some small plaques in two conditions of one of the 3 piglets. As illustrated in Figure 3, elastatinal did not reduce the plaque depth underneath the BM. The elastatinal wash-out experiment also did not have an effect on either plaque latitude or depth underneath the BM (data not shown).

## Discussion

Interactions of herpesviruses with respiratory mucosa remain poorly understood. Invasion of different alphaherpesviruses towards the lamina propria involves passage of the BM barrier. This facilitates viral access to blood capillaries and nerve endings which enables further dissemination throughout the body [2-12]. To date, the mechanism of the alphaherpesvirus invasion process through the BM barrier is unknown.

The current study shows that serine protease activity is involved in PRV invasion through the BM. Over one third of all known proteolytic enzymes are serine proteases which mediate diverse biological processes requiring timely and specifically regulated proteolytic activity, where insufficient or excessive proteolysis can cause pathological processes [22,23]. To date, knowledge of the role of serine proteases in the pathology of herpesvirus infections remains limited to the intracellular viral replication cycle itself. Proteolytic cleavage of viral proteins by cellular or viral proteases has been described in different stages of the herpesvirus life cycle [24]. An observation suggesting that proteases may modulate herpesvirus pathogenesis was made by Riteau et al. [25]. These authors suggest that herpesviruses can take advantage of mediators of inflammatory processes, namely extracellular proteases. Exposure of PRV-infected cells to trypsin increases virus production through activation of the extracellular signal-regulated kinase (ERK)1/2 signaling pathway. Reports describing protease-mediated extracellular matrix remodeling in association with viral infection are limited. Human papilloma virus (HPV) has been reported to induce both matrix metalloproteinase 2 (MMP-2) and MMP-9, two type IV collagenases. Type IV collagenases degrade the collagen type IV component of the basement membrane [26,27]. Two herpesviruses, Epstein-Barr virus (EBV) and herpes simplex virus (HSV), have also been reported to induce MMP-9 [14,28]. Whether this MMP-9 induction plays a role in EBV pathogenesis is unclear at present [28]. HSV encephalitis is characterized by an early MMP-9 increase and collagen type IV degradation causing disruption of the neurovascular matrix. This overexpression in proteolytic activity is believed to result from central nervous system inflammation, rather than from the viral infection itself and its role in pathogenesis is unclear [14].

More specifically, in the current study, we demonstrate that a trypsin-like serine protease is involved in viral penetration of the BM. Since serine protease activity involvement has been described in different stages of the herpesvirus life cycle [24], the effect of the Soybean trypsin-like protease inhibitor on general PRV replication was tested in ST and MDCK cells. No defect in viral replication was observed (data not shown), which is consistent with the observation that none of the inhibitors tested affected lateral viral spread above the BM in explants (Figures 1, 2, 3 and 4). Inhibition of trypsin-like serine protease activity strongly reduced the plaque depth underneath the BM. The time course of trypsin-like serine protease inhibitor treatment revealed less prominent reductions in plaque depth underneath the BM when explants were treated at later time points pi. These results are in line with our observation that PRV already crosses the BM between 12 and 16 h pi. In addition to trypsin-like proteases, serine proteases also encompass chymotrypsin-like and elastase-like proteases [29]. Therefore, a similar time course experiment was performed using inhibitors for chymotrypsin-like (TPCK) or elastase-like (elastatinal) serine proteases. No plaques were observed after treatment with TPCK, except for some small plaques in two conditions of one of the 3 piglets (3 h pi and 6 h pi). This perhaps may point to side or toxic effects of this inhibitor. Therefore, the involvement of chymotrypsin-like serine protease activity in PRV BM penetration could not be examined. Elastatinal did not reduce the plaque depth underneath the BM. However, since working solutions of elastase-like serine protease inhibitors have a relatively low stability (datasheet), we cannot formally exclude the possible involvement of either chymotrypsin-like or elastase-like serine protease activity in BM passage of PRV.

We can conclude that trypsin-like serine protease activity is involved in PRV invasion through the BM towards the lamina propria. We hypothesize that this trypsin-like serine protease activity is involved in local degradation of BM barrier components, thereby enhancing BM penetration. An important question that remains is whether the proteases involved are of cellular and/or viral origin. Interestingly, transcriptome studies have indicated that several cellular components involved in trypsin-like serine protease activity are differentially regulated upon infection with alphaherpesviruses like PRV and HSV, including factor IX, tissue-type plasminogen activator (t-PA) and urinary plasminogen activator receptor 1 (uPAR1) [30,31]. Further research will be aimed at identifying the proteases involved and elucidating the exact molecular mechanism of viral penetration through the BM.

## Competing interests

The authors declare that they have no competing interests.

## Authors' contributions

SG set up the study design, carried out the experiments, processed all samples, performed the statistical analysis and drafted the manuscript. HWF participated in the design of the study. LS and APV assisted in sampling. HJN took part in the design and coordination of the study. All authors read and approved the final manuscript.

## References

[B1] Knight DA Holgate ST The airway epithelium: structural and functional properties in health and disease Respirology 2003 8 432 446 10.1046/j.1440-1843.2003.00493.x 14708552

[B2] Engels M Ackermann M Pathogenesis of ruminant herpesvirus infections Vet Microbiol 1996 53 3 15 10.1016/S0378-1135(96)01230-8 9010994

[B3] Glorieux S Favoreel HW Meesen G de vos W Vanden Broeck W Nauwynck HJ Different replication characteristics of historical pseudorabies virus strains in porcine respiratory nasal mucosa explants Vet Microbiol 2009 136 341 346 10.1016/j.vetmic.2008.11.005 19111405

[B4] Kritas SK Pensaert MB Mettenleiter TC Role of envelope glycoproteins gI, gp63 and gIII in the invasion and spread of Aujeszky's disease virus in the olfactory nervous pathway of the pig J Gen Virol 1994 75 2319 2327 10.1099/0022-1317-75-9-2319 8077930

[B5] Kritas SK Pensaert MB Mettenleiter TC Invasion and spread of single glycoprotein deleted mutants of Aujesky's disease virus (PRV) in the trigeminal nervous pathway of pigs after intranasal inoculation Vet Microbiol 1994 40 323 334 10.1016/0378-1135(94)90120-1 7941296

[B6] Miry CM Pensaert MB van Oirschot JT Aujeszky's disease virus replication in tonsils and respiratory tract of non-immune and immune pigs Vaccination and control of Aujeszky's disease 1993 1 Dordrecht: Kluwer Academic Publishers 163 173

[B7] Nauwynck H Glorieux S Favoreel H Pensaert M Cell biological and molecular characteristics of pseudorabies virus infections in cell culture and in pigs with emphasis on the respiratory tract Vet Res 2007 38 229 241 10.1051/vetres:200661 17257571

[B8] Patel JR Heldens J Equine herpesviruses 1 (EHV-1) and 4 (EHV-4) - epidemiology, disease and immunoprophylaxis: A brief review Vet J 2005 170 14 23 10.1016/j.tvjl.2004.04.018 15993786

[B9] Pusterla N Wilson WD Madigan JE Ferraro GL Equine herpesvirus-1 myeloencephalopathy: A review of recent developments Vet J 2009 180 279 289 10.1016/j.tvjl.2008.08.004 18805030

[B10] Sabo A Rajcani J Blaskovic D Studies on the pathogenesis of Aujeszky's disease. I. Distribution of the virulent virus in piglets after peroral infection Acta Virol 1968 12 214 221 4385151

[B11] Sabo A Rajcani J Blaskovic D Studies on the pathogenesis of Aujeszky's disease. III. The distribution of virulent virus in piglets after intranasal infection Acta Virol 1969 13 407 414 4398706

[B12] Wittmann G Jakubik J Ahl R Multiplication and distribution of Aujeszky's disease (pseudorabies) virus in vaccinated and nonvaccinated pigs after intranasal infection Arch Virol 1980 66 227 240 10.1007/BF01314736 6160831

[B13] Buttle DJ Factors controlling matrix turnover in health and disease Biochem Soc Trans 2007 35 643 646 10.1042/BST0350643 17635111

[B14] Sellner J Simon F Meyding-Lamade U Leib SL Herpes-simplex virus encephalitis is characterized by an early MMP-9 increase and collagen type IV degradation Brain Res 2006 1125 155 162 10.1016/j.brainres.2006.09.093 17109833

[B15] Glorieux S Vanden Broeck W van der Meulen KM Van Reeth K Favoreel HW Nauwynck HJ In vitro culture of porcine respiratory nasal mucosa explants for studying the interaction of porcine viruses with the respiratory tract J Virol Methods 2007 142 105 112 10.1016/j.jviromet.2007.01.018 17324473

[B16] Nauwynck HJ Pensaert MB Abortion induced by cell-associated pseudorabies virus in vaccinated sows Am J Vet Res 1992 53 489 493 1316724

[B17] Hartley BS Proteolytic enzymes Annu Rev Biochem 1960 29 45 72 10.1146/annurev.bi.29.070160.000401 14400122

[B18] Barrett AJ Tolle DP Rawlings ND Managing peptidases in the genomic era Biol Chem 2003 384 873 882 10.1515/BC.2003.098 12887054

[B19] Walker B Lynas JF Strategies for the inhibition of serine proteases Cell Mol Life Sci 2001 58 596 624 10.1007/PL00000884 11361094 PMC11146489

[B20] Kunitz M Crystallization of a trypsin inhibitor from soybean Science 1945 101 668 669 10.1126/science.101.2635.668 17777539

[B21] Mignon B Swinnen M Bouchara JP Hofinger M Nikkels A Pierard G Gerday C Losson B Purification and characterization of a 315 kDa keratinolytic subtilisin-like serine protease from Microsporum canis and evidence of its secretion in naturally infected cats Med Mycol 1998 36 395 404 10206750

[B22] Hedstrom L Serine protease mechanism and specificity Chem Rev 2002 102 4501 4524 10.1021/cr000033x 12475199

[B23] Page MJ Di Cera E Serine peptidases: Classification, structure and function Cell Mol Life Sci 2008 65 1220 1236 10.1007/s00018-008-7565-9 18259688 PMC11131664

[B24] Jovasevic V Liang L Roizman B Proteolytic cleavage of VP1-2 is required for release of herpes simplex virus 1 DNA into the nucleus J Virol 2008 82 3311 3319 10.1128/JVI.01919-07 18216103 PMC2268474

[B25] Riteau B de Vaureix C Lefèvre F Trypsin increases pseudorabies virus production through activation of the ERK signalling pathway J Gen Virol 2006 87 1109 1112 10.1099/vir.0.81609-0 16603510

[B26] Katori H Nozawa A Tsukuda M Increased expression of matrix metalloproteinase-2 and 9 and human papilloma virus infection are associated with malignant transformation of sinonasal inverted papilloma J Surg Oncol 2006 93 80 85 10.1002/jso.20386 16353190

[B27] Smola-Hess S Pahne J Mauch C Zigrino P Smola H Pfister HJ Expression of membrane type 1 matrix metalloproteinase in papillomavirus-positive cells: role of the human papillomvirus (HPV) 16 and HPV8 E7 gene products J Gen Virol 2005 86 1291 1296 10.1099/vir.0.80551-0 15831939

[B28] Takeshita H Yoshizaki T Miller WE Sato H Furukawa M Pagano JS Raab-Traub N Matrix metalloproteinase 9 expression is induced by Epstein-Barr virus latent membrane protein 1 C-Terminal activation regions 1 and 2 J Virol 1999 73 5548 5555 10364303 10.1128/jvi.73.7.5548-5555.1999 PMC112612

[B29] Beynon RJ Bond JS Beynon RJ, Bond JS Proteolytic enzymes: A practical approach Appendix III 1989 Oxford University Press, Oxford, UK 244 246

[B30] Blanchard Y Le Meur N Le Cunff M Blanchard P Léger J Jestin A Cellular gene expression survey of pseudorabies virus (PRV) infected human embryonic kidney cells (HEK-293) Vet Res 2006 37 705 723 10.1051/vetres:2006027 16820135

[B31] Ray N Enquist LW Transcriptional response of a common permissive cell type to infection by two diverse alphaherpesviruses J Virol 2004 78 3489 3501 10.1128/JVI.78.7.3489-3501.2004 15016872 PMC371087

